# Transfersomal eosin topical delivery assisted by fractional CO_2_ laser for photodynamic treatment of palmar hyperhidrosis: case study

**DOI:** 10.1007/s13346-022-01164-z

**Published:** 2022-04-20

**Authors:** Doaa A. Abdel Fadeel, Maha Fadel, Abeer Tawfik, Yasser Omar

**Affiliations:** 1 Pharmaceutical Technology Unit, Department of Medical Applications of Laser, National Institute of Laser Enhanced Sciences (NILES), Cairo University, P.O. 12613, Giza, Egypt; 2 Dermatology Unite, Department of Medical Applications of Laser, National Institute of Laser Enhanced Sciences (NILES), Cairo University, P.O. 12613, Giza, Egypt; 3 Department of Pharmacy Practice and Clinical Pharmacy, Faculty of Pharmaceutical Sciences and Pharmaceutical Industries, Future University, Cairo, Egypt

**Keywords:** Hyperhidrosis, Photodynamic therapy, Eosin yellow photosensitizer, Transfersomes

## Abstract

Hyperhidrosis is a condition in which the cholinergic receptors on the eccrine glands are overstimulated, resulting in excessive sweating. It is considered a serious cosmetic and psychological problem that affects the patient’s quality of life. Searching for novel treatment modalities is required to minimize the side effects and to attain better patient satisfaction.

Photodynamic therapy (PDT), using eosin as a photosensitizer, is developed as a promising modality of the treatment of palmar and axillary hyperhidrosis. In this study, we treated six cases suffering palmar hyperhidrosis by applying the fractional CO_2_ laser prior to PDT session. For PDT, a hydrogel of eosin loaded in a transfersomes as a nano-delivery carrier was applied for 5 min, followed by irradiation by intense pulsed light (IPL). The prepared transfersomes loaded by eosin were spherical in shape with encapsulation efficiency of 33 ± 3.5%, particle size 305.5 ± 5.7 nm, average zeta potential of − 54 ± 7.6 mV with 80 ± 4% of the loaded eosin was released after 3 h. Two cases achieved 90% improvement after four sessions, three patients needed six sessions to show 75% improvement, while one patient showed only 25% improvement after six sessions. This resulted in shortening the time of PS application and decreasing the number of sessions required to achieve acceptable improvement. More clinical studies on large number of patients are required to optimize the results.

**Graphical abstract:**

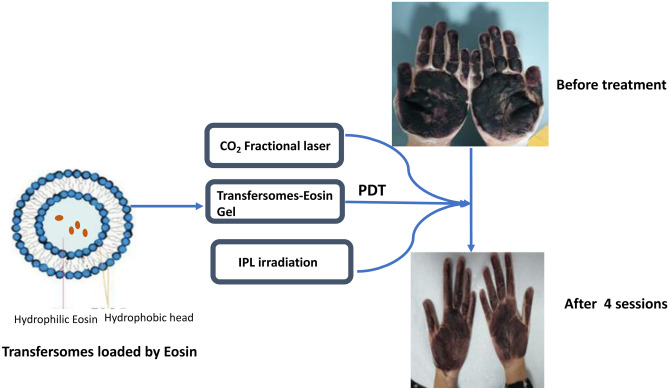

## Introduction

Hyperhidrosis is a condition in which the cholinergic receptors on the eccrine glands are overstimulated, resulting in excessive sweating. It could be either primary or secondary, can be generalized or localized in certain areas such as the axilla, feet, and palms [1]. Consequently, it is considered a serious cosmetic and psychological problem that affects the patient’s quality of life [2].

Several treatment strategies are available; however, most of them possess serious side effects. Topical therapy with antiperspirants and botulinum toxin may develop contact reactions and intolerable pain. Systemic therapy with anticholinergic drugs is associated with several systemic side effects such as mouth dryness, ocular, and GIT disorders. Surgical treatment is available but it is an invasive technique that lacks patient satisfaction [3, 4]. Therefore, searching for novel treatment modalities is required to overwhelm the aforementioned drawbacks.

Photodynamic therapy (PDT) is developed as a promising modality for the treatment of various malignant and non-malignant skin diseases. A chemical compound called photosensitizer (PS) is activated by light with a certain wavelength which excites the electrons of the PS. In the presence of oxygen, the excited molecule is reverted to its ground state by means of either two mechanisms: type I reaction producing free radicals (FR), or type II reaction producing reactive oxygen species (ROS) and singlet oxygen (^1^O_2_). The produced compounds are cytotoxic and result in the devastation of the target tissues. The PS itself is non-toxic, and it becomes toxic only upon radiation with an appropriate wavelength light source. The main advantage of the topical PDT is the specificity which is achieved by application of the PS to the desired site of action and the irradiation of this site only. Consequently, the production of FR, ROS, and /or ^1^O_2_ is confined only to the diseased tissue, while the normal tissues remained unaffected [5].

In topical PDT, the *stratum corneum* (SC) is the main obstacle that prevents PS penetration, especially hydrophilic ones. There are many physical approaches to overcome the SC barrier properties, such as ultrasound, iontophoresis, microneedles, and electroporation. These techniques are invasive, need sophisticated devices, and have many limitations in the clinical applications [6, 7].

Nanotechnology has emerged as a strategy to facilitate dermal and transdermal drug delivery. Encapsulating PS in a lipid nano-vesicular system such as liposomes, transfersomes, niosomes, and ethosomes could be a promising approach to enhance the passage of the PS through the SC, hence improving the penetration of drugs to deep skin layers [8].

The proper choice of the PS and the use of the appropriate delivery system to carry it across the SC are crucial to enhance the topical PDT efficacy.

Eosin yellow (EY) is a hydrophilic dye that was clinically approved for many clinical applications and exhibited marked efficiency as a photosensitizer in PDT of many skin disorders [9]. PDT using EY has been used successfully for the treatment of axillary hyperhidrosis by the author [10] and for the treatment of palmar hyperhidrosis [11]; however, palmar hyperhidrosis is still challenging due to the increased thickness of the palmar skin.

Regarding the delivery system, transfersomes have been widely studied as an efficient delivery system to enhance the topical application of PS. They are phospholipid bilayer vesicles with an edge activator in their membrane. The lipidic membrane imparts the required hydrophobicity to penetrate through the SC, while an edge activator, a surfactant, imparts the required elasticity and deformability to the vesicle membrane [12, 13].

In previous work, we succeeded to treat plantar warts by topical PDT using EY loaded in transfersomes [14]. This encouraged us to use this formula to treat another challenging skin disorder, palmar hyperhidrosis. Moreover, in this study, we used a CO_2_ fractional laser prior to the application of EY to facilitate its penetration, hence improving the clinical outcome of the PDT. Collectively, in this study, we provided a new protocol for the treatment of palmar hyperhidrosis. This protocol combines the fractional CO_2_ laser and PDT using EY loaded in transfersomes as a nano-delivery carrier.

## Materials

Eosin Y (EY) 90% dye content (Mwt 691.88) was purchased from Loba Chemie, India. Sodium deoxycholate (SDC), lecithin from soybean oil, phosphate-buffered saline (PBS), and chloroform were purchased from Sigma Aldrich. Carboxymethyl cellulose Na (Na-CMC) was purchased from Normest Company, Egypt, for scientific development. Methanol and absolute ethanol (99%) were purchased from El Nasr Pharmaceutical Chemicals Co., Adwic, Egypt.

## Methods

### Preparation of transfersomes loaded by eosin

Transfersomes loaded by EY were prepared as previously described in our previous work [14]. Briefly, the lipid (lecithin from soybean oil) and the edge activator (sodium deoxycholate) were dissolved in a mixture of chloroform: methanol 2:1. The organic solvents were then evaporated under vacuum in a rotary evaporator (Heidolph-Elektro GmbH + Co KG, Germany) rotating at 90 rpm at 45 °C. The rotation is continued till complete evaporation of the solvents and the formation of a uniform thin lipid film. The obtained lipid film was then hydrated by phosphate-buffered EY solution (PBS-EY) and left in the rotary evaporator for a further 1 h. The lipid: edge activator: EY ratio was set as 10:1:1. The transfersomes dispersion was sonicated for 5 min in a water bath sonicator and was stored in the refrigerator for further use.

### Characterization of the prepared transfersomes

#### I. Morphology and shape

The diluted transfersomal dispersion was examined by a fluorescence microscope (Olympus BX51), supplied by a 75-W xenon lamp as an excitation source.

Moreover, the shape of the prepared transfersomes was examined under transmission electron microscopy (TEM, Jeol, Ltd., Tokyo, Japan) after negative staining.

#### II. UV–visible spectroscopy

The absorption spectrum of (PBS-EY) solution and EY-transfersomes dispersion were recorded by double beam spectrophotometer (RayLeigh UV-2601) at wavelength range 400–700 nm, using PBS solution as a blank reference.

#### III. Encapsulation efficiency (EE)

The unloaded EY was separated from the loaded transfersomes by centrifugation at 10,000 rpm at 5–6 °C (Centrikon T-42 K, Kontron Instruments, UK). The precipitated loaded transfersomes were dissolved in ethanol, to extract EY from them, and diluted by PBS (pH 7.4). The concentration of the extracted EY was measured spectrophotometrically at 520 nm (Rayleigh UV-2601) from a previously established standard calibration curve in PB-ethanol solution. Finally, the EE was determined as the following: EE % = (the calculated EY concentration extracted from the transfersomes/initial EY concentration used in preparation) X100.

#### IV. Particle size and zeta potential

The mean particle size and zeta potential of the prepared transfersomes were measured by the Zetasizer system (Malvern Instruments Ltd., Malvern, UK) after ten folds dilution with double distilled water at laser wavelength 632 nm, a temperature of 23 °C, scattering angle of 14.1°, and a refractive index of 1.33.

#### V. In-vitro release of EY from the prepared transfersomes

Three aliquots (*n* = 3) of the transfersomes suspension were dispersed in PBS (pH 7.4) and incubated separately at 37 °C under continuous stirring in a water bath. Each of the incubated dispersions was centrifuged for 10 min at 10,000 rpm at preset intervals. The precipitates were re-dispersed in fresh PBS and the concentration of released EY in the supernatants was measured by measuring the absorbance at 520 nm. Finally, the cumulative EY released percentage was determined as a function of time.

### Preparation of transfersomal eosin hydrogel

The prepared EY-loaded transfersomes were incorporated into 5% Na-CMC hydrogel. A volume of transfersomal suspension, corresponding to 20 mg EY, was completed to 100 ml distilled water. Then, 5 gm of Na-CMC was added portion-wise with continuous stirring till full dispersion. Methylparaben (0.1%) and propylparaben (0.2%) were then added with continuous stirring to serve as preservatives. The gel was left for 24 h for complete swelling and to ensure the absence of any aggregates or air bubbles.

### Clinical cases

#### Patients

The study was conducted at the dermatology clinic at the National Institute of Laser Enhanced Sciences (NILES), Cairo University, Egypt, after the approval of the board of the research ethics committee of the Institute (approval no: CU-NILES/24/21). The study protocol conformed to the guidelines of the Declaration of Helsinki. This case study was conducted on 6 patients, representing 6 case reports. The demographic data of the patients are listed in Table 1. A written informed consent was obtained from every patient. The authors affirm that one of the participants provided informed consent for publication of the images in Fig. 4a and b.

**Table 1:** Patients characteristic

**Patients’ characteristics**	**Number/percentage**
Number of patients	6 patients
Sex	Females
Site	Bilateral palmar hyperhidrosis
Age	Range: 20–38 years (23,20,21,22,38,21)
Duration of suffering hyperhidrosis	Range: 2–6 years (3,2,5,3,2,3)
Affecting factors	Hot weather in 3, stress in 3 patients

Exclusion criteria included children, pregnant or lactating females.

#### Treatment protocols

The palms were subjected to 2 passes of fractional CO_2_ laser (Bison, Italy) with the following parameters; scanning area 3 ~ 20 mm, Y: 3 ~ 20 mm, the fluence 7.5 mJ/cm^2^, pulse duration 500 µs and dot density 0.8. Afterward, the transfersomal EY gel was applied for 5 min.

The affected sites were then irradiated using an intense pulsed light (IPL, EPI-C PLUS; Espansione Group, Bologna, Italy), as a light source to activate the photosensitizer, fitted with a 550 nm filter that matches the maximum absorption peak of EY (2.5 × 4.5 cm spot area, total area 11.25 cm^2^, 20 ms pulse duration, and 25 J/cm^2^ fluence). Sessions were performed once per week, for 6 weeks. The assessment was done by starch iodine test to determine the area of hyperhidrosis before and after treatment. The sweating intensity was determined based on the color intensity of the starch iodine test according to the 5-grades Sweating Intensity Visual Scale [11], where Grade 0 indicates no sweating, grade 1 indicates minimal sweating, grade 2 indicates mild sweating, grade 3 indicates moderate sweating, grade 4indicates intense sweating, and grade 5 indicates over-sweating.

Moreover, patient satisfaction was measured by a scoring system: 0 (unsatisfied), 1 (partially satisfied), and 2 (very satisfied).

## Results and discussion

### Preparation and characterization of transfersomes loaded by eosin

**Fig. 1:**
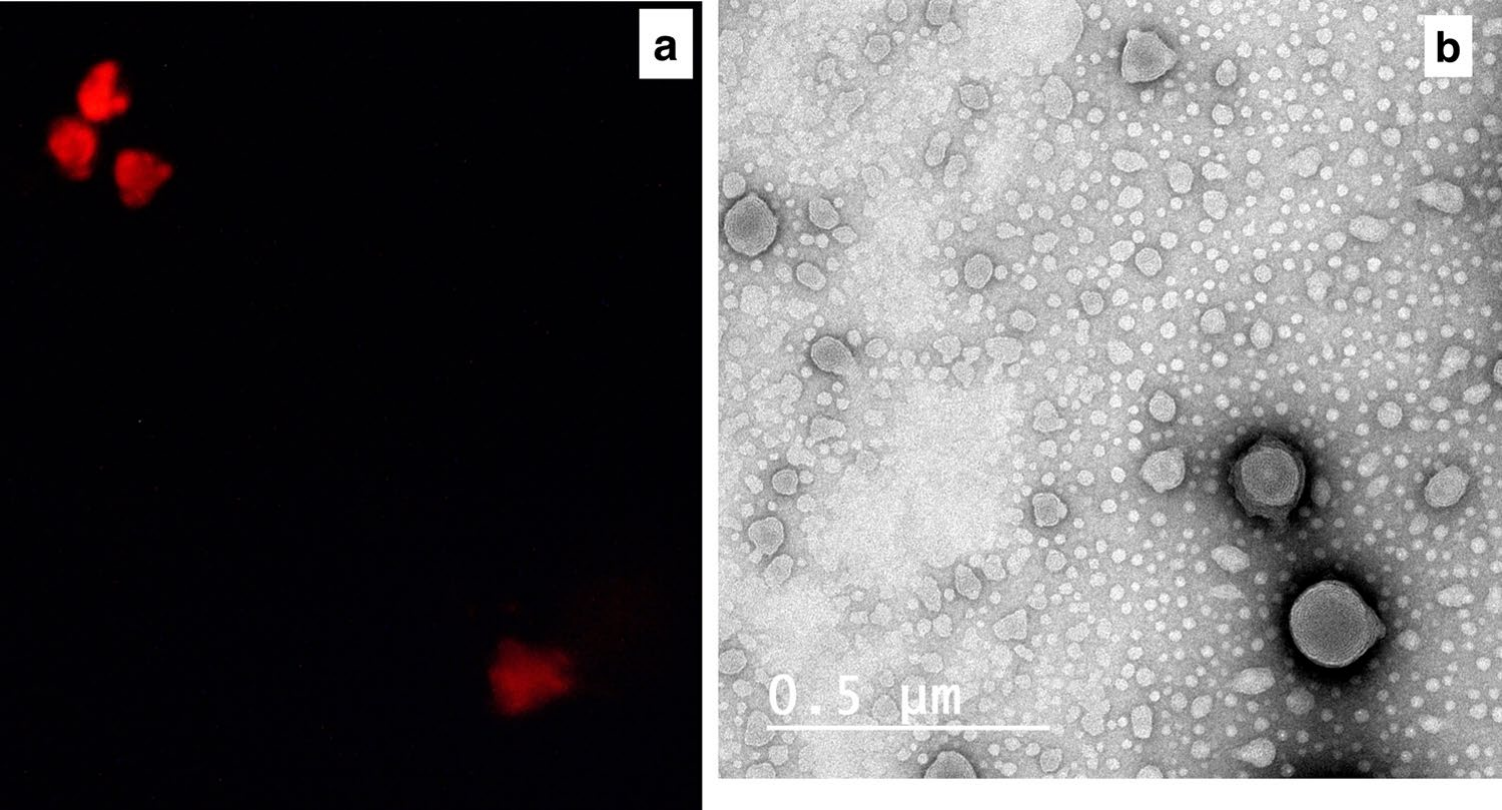
(**a**) Fluorescence microscope micrograph and (**b**) TEM image of the prepared eosin-loaded transfersomes

Transfersomal vesicles loaded by EY were spontaneously formulated upon hydration of the lipid film with PBS-EY solution. Being a hydrophilic drug, EY was incorporated in the aqueous core of the transfersomal vesicles, as indicated by the fluorescence micrograph (Fig. 1a). TEM image (Fig. 1b) proved the formation of multilayered spherical vesicles with no aggregation or irregularities.

**Fig. 2:**
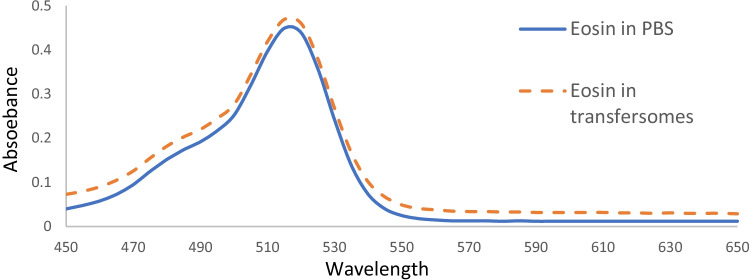
Spectroscopic properties of eosin in PBS solution and in the prepared transfersomes

The peaks of the maximum absorption of both PBS-EY and EY loaded in transfersomes were found to be at 520 nm (Fig. 2), indicating that the loading of EY in transfersomes did not affect its spectroscopic properties.

The calculated EE was 33 ± 3.5% which is considered high for a hydrophilic drug as EY.

**Fig. 3:**
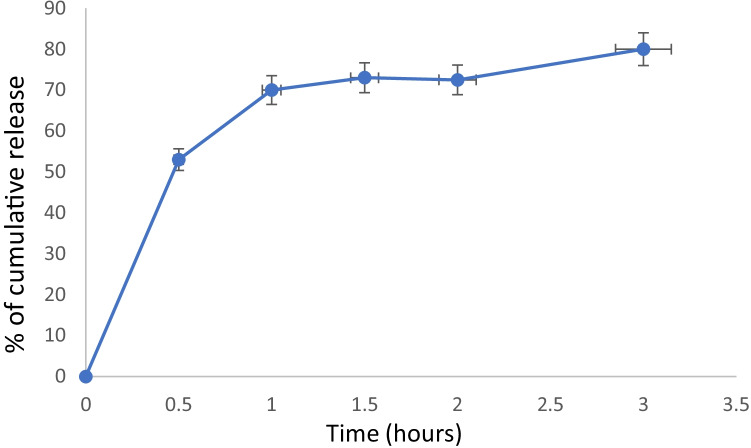
In vitro release of eosin from the prepared transfersomes

The mean diameter was found to be 305.5 ± 5.7 nm with a polydispersity index (P.I.) of 0.5 indicating homogenous particle size distribution. The prepared transfersomes exhibited an average zeta potential of − 54 ± 7.6 mV indicating good stability. The release profile (Fig. 3) showed that 80 ± 4% of EY was released from the transfersomes after 3 h.

Transfersomes are lipid vesicles that resemble the liposomes in their structure, but their lipid membrane’s composition is altered by the addition of an edge activator[12].

Previous studies on hyperhidrosis treatment used liposomes, to enhance the drug delivery through palmar skin [11, 15]. In this case study, we used transfersomes as it was proved that transfersomes were superior to liposomes in the dermal and transdermal delivery [16]. The presence of edge activator, as SDC, in their lipid membrane improved the membrane deformability and flexibility, permits the passage of the vesicles between the narrow skin pores, thus delivering the drug to deeper skin layers without being cracked [17].

### Clinical cases

The results of our case studies revealed that two patients achieved 90% improvement after four sessions, three patients needed six sessions to show 75% improvement, and one patient showed only 25% improvement after six sessions (Table 2). The improvement was determined based on the sweating intensity visual scale and the patient satisfaction scoring system.

**Table 2:** Patient’s outcome

**Degree of improvement**	**Number of sessions**	**Relapse**
3 patients: 75% 2 patients: 90% 1 patient: 25%	6 sessions 4 sessions 6 sessions	- - After 1 year

**Fig. 4:**
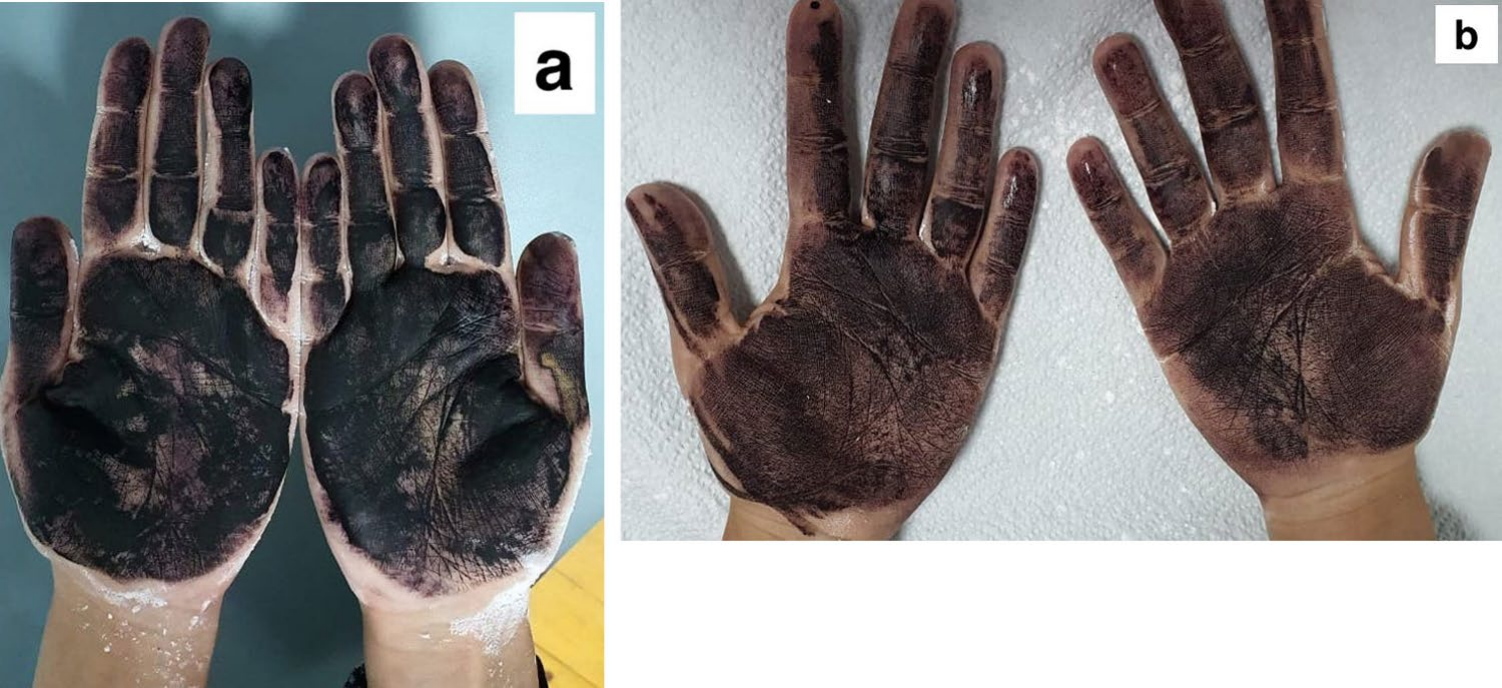
(**a**) Iodine starch test before treatment showing dark blue color and (**b**) iodine starch test after treatment showing light blue color

There was a marked improvement as stated by the patients (80% at the palm and 70% at the fingers, Fig. 4a, b).

The pain experienced throughout the treatment sessions was manageable, and no topical anesthetics were required.

The treatment mechanism of hyperhidrosis by PDT relies on ROS production upon excitation of the PS by light, thus affecting the eccrine glands and reducing their sweat production [10].

PDT using EY as a PS (PDT-EY) was used previously for the treatment of both axillary [10] and palmar hyperhidrosis [11]. The results obtained from those studies revealed that the management of palmar hyperhidrosis was more difficult and took a longer time and higher number of sessions (8 sessions). Moreover, the EY gel was applied for 1 h prior to irradiation, which caused patients dissatisfaction. This is because the palms are characterized by thick skin, thus it is difficult for the topically applied drug to reach the eccrine glands.

However, in this case study, the patients needed a smaller number of sessions (4–6 sessions) with no relapse (Table 2). We applied the EY transfersomal gel for 5 min only, which was more convenient to the patients.

This may be attributed to two reasons; the first is the loading of EY in transfersomes which facilitate the passage and the penetration of EY through the palmar skin as described above. The second reason is the combination between CO_2_ fractional laser and PDT.

CO_2_ fractional laser was used as trans-epidermal drug delivery to facilitate the penetration of the PS. It causes ablation of the upper skin layer creating minute vertical channels that extend to the upper dermis. Through these channels, the topically applied drugs can penetrate to deeper epithelial layers [18]. It has been reported that the use of CO_2_ fractional laser prior to PDT was beneficial in the treatment of actinic keratoses [19, 20], high-risk basal cell carcinomas [21], Bowen disease [22], onychomycosis [23], and actinic cheilitis [24]. However, the combination between CO_2_ fractional laser and PDT did not impart a significant effect in the treatment of vulvar Paget’s disease [25]. All of the aforementioned studies were done using 5-aminolevulinic acid and its derivative methyl aminolevulinate as a photosensitizer. Up to our knowledge, no previous studies investigated the combined effect of CO_2_ fractional laser with EY-PDT for the treatment of palmar hyperhidrosis.

## Conclusion

Continuous endeavors for the treatment of hyperhidrosis revealed that PDT can be a promising modality of treatment. In this study, we tried to improve the clinical outcome of PDT treatment by two approaches. The first one is the loading of EY (PS) in transfersomes which are deformable vesicles that improve penetration of drugs through skin layers. The second one is the use of a CO_2_ fractional laser prior to PDT treatment to assist the penetration of the photosensitizer. This resulted in shortening the time of PS application and decreasing the number of sessions required to achieve acceptable improvement. More clinical studies on a larger number of patients are required to optimize the results.

## Data Availability

All data can be available.
